# Wdr66 is a novel marker for risk stratification and involved in epithelial-mesenchymal transition of esophageal squamous cell carcinoma

**DOI:** 10.1186/1471-2407-13-137

**Published:** 2013-03-21

**Authors:** Qing Wang, Chenming Ma, Wolfgang Kemmner

**Affiliations:** 1 Experimental Clinical Research Center at the Max-Delbrueck-Center for Molecular Medicine, Charité Campus Buch, Lindenbergerweg 80, Berlin, 13125, Germany; 2 Charite Comprehensive Cancer Center, Charité Campus Mitte, Berlin, D-10115, Germany

**Keywords:** WD repeat-containing protein, Esophageal squamous cell carcinoma, Epithelial-mesenchymal transition

## Abstract

**Background:**

We attempted to identify novel biomarkers and therapeutic targets for esophageal squamous cell carcinoma by gene expression profiling of frozen esophageal squamous carcinoma specimens and examined the functional relevance of a newly discovered marker gene, WDR66.

**Methods:**

Laser capture microdissection technique was applied to collect the cells from well-defined tumor areas in collaboration with an experienced pathologist. Whole human gene expression profiling of frozen esophageal squamous carcinoma specimens (n = 10) and normal esophageal squamous tissue (n = 18) was performed using microarray technology. A gene encoding WDR66, WD repeat-containing protein 66 was significantly highly expressed in esophageal squamous carcinoma specimens. Microarray results were validated by quantitative real-time polymerase chain reaction (qRT-PCR) in a second and independent cohort (n = 71) consisting of esophageal squamous cell carcinoma (n = 25), normal esophagus (n = 11), esophageal adenocarcinoma (n = 13), gastric adenocarcinoma (n = 15) and colorectal cancers (n = 7). In order to understand WDR66’s functional relevance siRNA-mediated knockdown was performed in a human esophageal squamous cell carcinoma cell line, KYSE520 and the effects of this treatment were then checked by another microarray analysis.

**Results:**

High WDR66 expression was significantly associated with poor overall survival (P = 0.031) of patients suffering from esophageal squamous carcinomas. Multivariate Cox regression analysis revealed that WDR66 expression remained an independent prognostic factor (P = 0.042). WDR66 knockdown by RNA interference resulted particularly in changes of the expression of membrane components. Expression of vimentin was down regulated in WDR66 knockdown cells while that of the tight junction protein occludin was markedly up regulated. Furthermore, siRNA-mediated knockdown of WDR66 resulted in suppression of cell growth and reduced cell motility.

**Conclusions:**

WDR66 might be a useful biomarker for risk stratification of esophageal squamous carcinomas. WDR66 expression is likely to play an important role in esophageal squamous cell carcinoma growth and invasion as a positive modulator of epithelial-mesenchymal transition. Furthermore, due to its high expression and possible functional relevance, WDR66 might be a novel drug target for the treatment of squamous carcinoma.

## Background

Esophageal squamous cell carcinoma (ESCC) is one of the most lethal malignancies of the digestive tract and in most cases the initial diagnosis is established only once the malignancy is in the advanced stage [1]. Poor survival is due to the fact that ESCC frequently metastasizes to regional and distant lymph nodes, even at initial diagnosis.

Treatment of cancer using molecular targets has brought promising results and attracts more and more attention [2-5]. Characterization of genes involved in the progression and development of ESCC may lead to the identification of new prognostic markers and therapeutic targets.

By whole genome-wide expression profiling, we found that WD repeat-containing protein 66 (WDR66), located on chromosome 12 (12q24.31), might be a useful biomarker for risk stratification and a modulator for epithelial-mesenchymal transition of ESCC.

WD-repeat protein family is a large family found in all eukaryotes and is implicated in a variety of functions ranging from signal transduction and transcription regulation to cell cycle control, autophagy and apoptosis [6]. These repeating units are believed to serve as a scaffold for multiple protein interactions with various proteins [7]. According to whole-genome sequence analysis, there are 136 WD-repeat proteins in humans which belong to the same structural class [8]. Among the WD-repeat proteins, endonuclein containing five WD-repeat domains was shown to be up regulated in pancreatic cancer [9]. The expression of human BTRC (beta-transducing repeat-containing protein), which contains one F-box and seven WD-repeats, targeted to epithelial cells under tissue specific promoter in BTRC deficient (−/−) female mice, promoted the development of mammary tumors [10]. WDRPUH (WD repeat-containing protein 16) encoding a protein containing 11 highly conserved WD-repeat domains was also shown to be up regulated in human hepatocellular carcinomas and involved in promotion of cell proliferation [11]. The WD repeat-containing protein 66 contains 9 highly conserved WD40 repeat motifs and an EF-hand-like domain. A genome-wide association study identified a single-nucleotide polymorphism located within intron 3 of WDR66 associated with mean platelet volume [12].

WD-repeat proteins have been identified as tumor markers that were frequently up-regulated in various cancers [11,13,14]. Here, we report for the first time that WDR66 might be an important prognostic factor for patients with ESCC as found by whole human gene expression profiling. Moreover, to our knowledge, the role of WDR66 in esophageal cancer development and progression has not been explored up to now. To this end we examined the effect of silencing of WDR66 by another microarray analysis.

In addition, the effect of WDR66 on epithelial-mesenchymal transition (EMT), cell motility and tumor growth was investigated.

## Methods

### Patients

Tissue samples from individuals of the first set (n = 28) were obtained from the tumor bank of Charité Comprehensive Cancer Center. Gene expression was examined by whole-human-genome microarrays (Affymetrix, Santa Clara, CA, USA). Ten esophageal squamous cell carcinoma (ESCC) and 18 normal esophageal (NE) biopsies were randomly collected. Normal healthy esophageal biopsies were collected from patients with esophageal pain but diagnosed as normal squamous without pathological changes. Surgical specimens of chemotherapy-naïve patients with known ESCC of histological grading G1, UICC stage II and III, had undergone esophagectomy. Patients’ age ranged from 22 to 83 years, with a median age of 59 years.

A second panel (n = 71) consisting of ESCC (n = 25), NE (n = 11), esophageal adenocarcinoma (EAC) (n = 13), gastric adenocarcinoma (GAC) (n = 15) and colorectal cancers (CRC) (n = 7) was used for qRT-PCR validation. Patients’ age ranged from 24 to 79 years, with a median age of 63 years.

All samples were snap-frozen in liquid nitrogen and stored at −80°C. We obtained tissue specimens from all subjects with informed written consent (approved by the local ethics committee of the Charité-Universitätsmedizin, Berlin). Each single specimen included in this study was histopathologically approved according to grade and stage by an experienced pathologist ( MV, University Bayreuth).

### Laser capture microdissection and microarray

Laser Capture Microdissection (Cellcut, MMI AG and Nikon TE300 microscope) was used for isolating desired cells from sections. After transferring 5 μm sections onto MMI membrane slides, these were fixed in 70% isopropyl alcohol and then stained with the MMI basic staining kit. Desired tumor cell or NE areas were selected, cut and collected. Preparation of labeled cRNA and hybridization were done using the gene chip hybridization, wash, and stain kit (Affymetrix, Santa Clara, CA, USA), as described previously [15]. Two cycle labeling was applied to all samples. In total 28 chip-data were collected using Gene Chip Operation Software (GCOS, Affymetrix). The 28 specimens analyzed consists 10 ESCC and 18 NE. To obtain the relative gene expression measurements, probe set-level data extraction was performed with the GCRMA (Robust Multiarray Average) normalization algorithm implemented in GeneSpring GX10.2 (Agilent). All data were log2 transformed. A list of all the genes included in these microarrays and the normalized data have been deposited in the Gene Expression Omnibus database (http://www.ncbi.nlm.nih.gov/geo/info/linking.html) under GEO accession number GSE26886. For gene-by-gene statistical testing, parametric tests were used to compare differences between groups. The False Discovery Rate (FDR) was employed using Benjamini-Hochberg procedure for multiple testing of the resulting p-value significance.

### In situ hybridization

A 148 bp fragment located at the 3 terminal end of human WDR66 gene (NM144668)was subcloned into the pBluescript II vector pBS-27.16 using primer pair forward: 5’-CAACCTgCTCCgTCAAA-3’ and reverse: 5’-TAAACATTCCTggTAACTTCAC-3’. The linearized plasmid was used as a template for the synthesis of antisense probes. The probe was labeled by digoxigenin / dUTP with a DIG/RNA labelling kit (Boehringer Mannheim), according to the manufacturer’s instructions. The quality and quantity of the probe were confirmed by gel electrophoresis before used for In situ hybridization. The Digoxigenin-labeled probe was applied to 5 μm dewaxed FFPE sections and hybridized at 65°C overnight in a humid chamber. After 3 washes to remove the nonspecific binding or unbound probes, digoxigenin-labeled probe was detected using the alkaline phosphatase method.

### RNA extraction and qRT-PCR

RNA extractions were carried out using the RNeasy Mini Kit (Qiagen, Valencia, CA, USA). Total RNA quality and yield were assessed using a bioanalyzer system (Agilent 2100 Bioanalyzer; Agilent Technologies, Palo Alto, CA, USA) and a spectrophotometer (NanoDrop ND-1000; NanoDrop Technologies, Wilmington, DE, USA). Only RNA with an RNA integrity number > 9.0 was used for microarray analysis.

For quantification of mRNA-expression, qRT-PCR was performed for 3 genes plus one control, using pre-designed gene-specific TaqMan® probes and primer sets purchased from Applied Biosystems (Hs01566237_m1 for WDR66, Hs00958116_m1 for VIM, Hs00170162_m1 for OCLD, and 4326317E for GAPDH). Conditions and data analysis of qRT-PCR were as described [16].

### Cell culture and siRNA-mediated knockdown

KYSE520, OE33, SW480, Caco2, HCT116, HT29, HL60, LS174T, Daudi, HEK293, MCF7, MDA-MB-231, MDA-MB-435 and Capan-I were obtained from the American Type Culture Collection (ATCC, Manassas, VA) and cultured according to the supplier’s instructions. For siRNA-mediated knockdown of WDR66, cells were seeded in 6-well plates on the day before transfection. On day 0, cells were transfected with siRNA at 25 nmol/L concentrations using Thermo Scientific DharmaFECT transfection reagents according to manufacturer’s instructions. The siRNA sense 5’ – GuuACuAAAGGuGAGCAuA - 3’ sequence corresponding to WDR66 mRNA was chemically synthesized by sigma-Proligo (Munich, Germany). RNA was extracted at indicated time points as described.

### Microarray analysis of WDR66 knock-down cells

Total RNA was extracted from 10^6^ cell pellet using RNeasy Mini Prep Kit (Qiagen, Hilden, Germany). RNA quality was checked by Bioanalyzer (Agilent, Santa Clara, CA) Only RNA samples showing a RIN of at least 9.0 were used for labelling. Total RNA (1 μg) was labelled with Cy3 using the Low Input RNA Amplification Kit (Agilent, Santa Clara, CA). Labelled cRNAs were hybridized to Whole Human Genome 4x44K Oligonucleotide Microarrays (Agilent, Santa Clara, CA) according to the manual. Arrays were scanned by using standard Agilent protocols and a G2565AA Microarray Scanner (Agilent, Santa Clara, CA). Raw expression values were determined using Feature Extraction 9.0 software (Agilent, Santa Clara, CA).

### Western blotting analysis

Total cell extracts were obtained and cell lysate containing 50 μg of protein was separated on 10% SDS-polyacrylamide gel and then blotted onto polyvinylidene difluoride (PVDF) membranes (Millipore, Bedford, MA, USA). Primary antibody for vimentin detection was mouse monoclonal anti-human vimentin antibody (Sigma-Aldrich Corporation, V5255, 1:200, approximately 54 kDa). Primary antibody for occludin detection was rabbit polyclonal anti-human occludin antibody (Invitrogen, 71–1500, 1:500, at 65 kDa). ß-actin was used as loading control (Abcam, 1:2000, ab8226). Signals were detected using ECL kit (Amersham Pharmacia Biotech, Piscataway, NJ, USA). Images were scanned by FujiFilm LAS-1000 (FujiFilm, Düsseldorf, Germany).

### Cell number, cell motility and wound-healing assay

Cells were seeded in 6-well plates 24 h before transfection. Transfection was done as described above. Cells were collected at indicated time points, and cell numbers were measured using POLARstar Omega reader (BMG Labtech, Offenburg, Germany). Emission and excitation filters were 485 and 520 nm. The results were analyzed by MARS data analysis software. Cell motility was determined using 12-well Transwell Permeable Support inserts with polycarbonate filters with a pore size of 8 μm (Corning Costar, Lowell, MA), according to the manufacturer’s instructions. Wound healing assays were performed in triplicates using cytoselect 24-well wound healing assay (Cell Biolabs, Inc.).

### Statistical analysis

Statistical analysis was done using GraphPad Prism version 5 for Windows (GraphPad Software) and SPSS version 13 for Windows (SPSS, Chicago, IL, USA) as follows: GraphPad Prism, Unpaired t test with Welch’s correction of quantitative real-time RT-PCR measurements of WDR66 in patient samples and of gene expression measurements in the validation cohort; nonparametric Mann–Whitney U test of cell numbers, motility assay, and cell wound assay after knockdown of WDR66; SPSS, Kaplan-Meier survival analysis and log-rank statistics, cut-point analysis of qRT-PCR measurements of WDR66 in patient samples using maximally selected rank statistics to determine the value separating a group into two groups with the most significant difference when used as a cut-point; grouping of patients according to median of qRT-PCR measurements was done as follows: WDR66 ≤ 125, WDR66 low; WDR66 > 125, WDR66 high; the stratified Cox-regression model was used to determine prognostic factors in a multivariate analysis with WDR66 dichotomized at the previously determined cut-points.

## Results

### WDR66 is specifically highly expressed in esophageal squamous cell carcinoma

**Figure 1:**
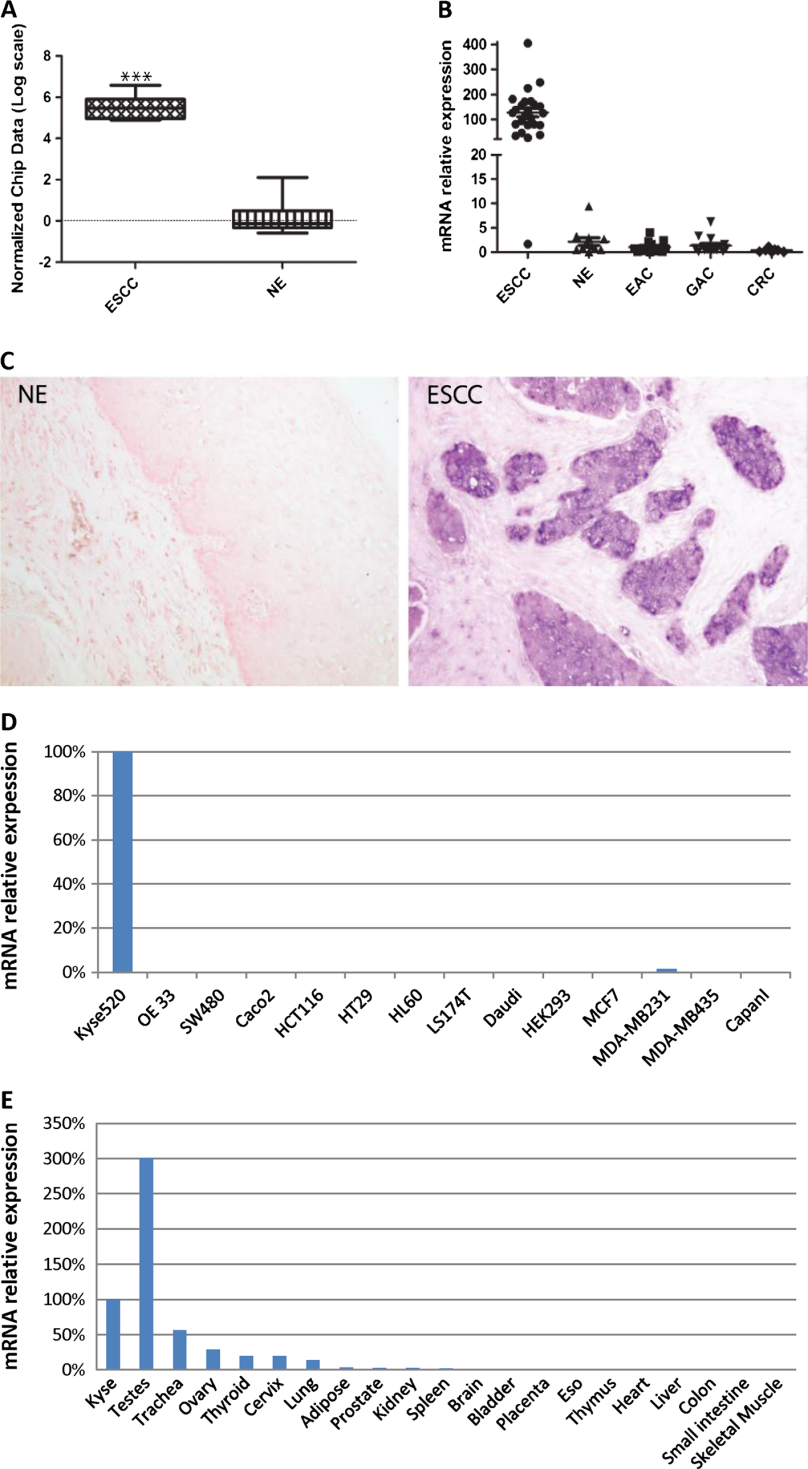
**WDR66 is highly expressed in esophageal squamous cell carcinoma. A**: mRNA expression of the WDR66 gene was determined by microarray analysis. Microarray analysis was performed on 18 healthy normal esophageal epithelium (NE) and 10 esophageal squamous cell carcinoma (ESCC) samples. Gene expression is presented as normalized (log2 scale) signal intensity of the WDR66 gene. The WDR66 gene is significantly differentially expressed in ESCC (corrected p-value < 0.0001). Expression level of WDR66 gene is low in NE but high in ESCC cases. **B**: Relative mRNA expression of the WDR66 gene in an independent validation cohort. Quantitation was done relative to the transcript of GAPDH. Significance in differential expression of individual gene between groups was calculated (p-value < 0.001). Results showed that WDR66 gene expression level is highest in ESCC and low to absent in NE or other carcinomas. On the horizontal axis patient groups ESCC, NE, EAC, GAC and CRC are depicted. **C**: WDR66 gene is highly expressed in ESCC epithelium according to in situ hybridization. In situ hybridization was down using anti-sense probes of human WDR66 gene in FFPE sections of esophageal specimens. Signals for WDR66 transcripts were observed specifically in esophageal squamous cell carcinoma (ESCC, right), but not in normal squamous epithelium (NE, left). **D**: WDR66 expression level in various human cell lines. WDR66 expression was examined by quantitative real-time PCR in 14 cell lines cultivated from different human carcinomas. The expression was quantified relative to human esophageal squamous carcinoma cell line KYSE520. **E**: Tissue-specific expression of WDR66 gene in various human normal tissues. Quantitative real-time PCR analysis of WDR66 expression levels in 20 human normal tissues (FirstChoice® Human Total RNA Survey Panel). WDR66 gene is preferentially expressed in testis. Gene level was quantified relative to the expression in ESCC cell line KYSE520.

Whole genome-wide expression profiling was performed on 28 esophageal specimens (GSE26886). To make sure that only epithelial cells were studied, we applied laser capture microdissection technique to the specimens. A number of genes differentially expressed between ESCC samples and normal esophageal squamous epithelium samples were identified. The probe set with the highest fold change and lowest p-value represented the WDR66 transcript (P < 0.0001) (Figure 1A). As a validation study, WDR66 expression was examined by qRT-PCR in an independent cohort consisting of 71 specimens including ESCC (n = 25), NE (n = 11), EAC (n = 13), GAC (n = 15) and CRC (n = 7). We found that WDR66 was highly expressed in 96% of ESCC patients (Figure 1B). Confirming our previous results from the microarray study, WDR66 expression was found to be significantly higher in ESCC compared to NE as well as the other three cancer types checked in this cohort (P < 0.0001). Immunohistochemical localization of WDR66 was not carried out because none of the WDR66 antibodies available allowed detecting a specific protein band on Western blots. The presence of WDR66-specific mRNA was probed by in-situ hybridization using single-stranded RNA probes of the WDR66 gene in 4% PFA-fixed paraffin-embedded esophageal tissues. WDR66 transcription (positive staining) was specifically detected in the esophageal squamous carcinoma cells but not in normal squamous epithelia (Figure 1C). Furthermore, WDR66 expression was examined in 14 human cell-lines and 20 normal human tissues by qRT-PCR. Expression of WDR66 gene was abundantly expressed only in the human esophageal squamous cell carcinoma cell line KYSE520, but not expressed in any other human cell line, such as OE33, SW480, HT29, HCT116, LS174T, Caco2, HL60, HEK293, Daudi, Capan1, MCF7, MDA-MB231 and MDA-MB435 (Figure 1D). Among 20 normal human tissues examined by qRT-PCR, WDR66 was most abundantly expressed in the testis (Figure 1E). Thus, our data suggest that WDR66 might be a cancer / testis antigen.

### High expression of WDR66 correlates with poor survival outcome in ESCC

**Figure 2:**
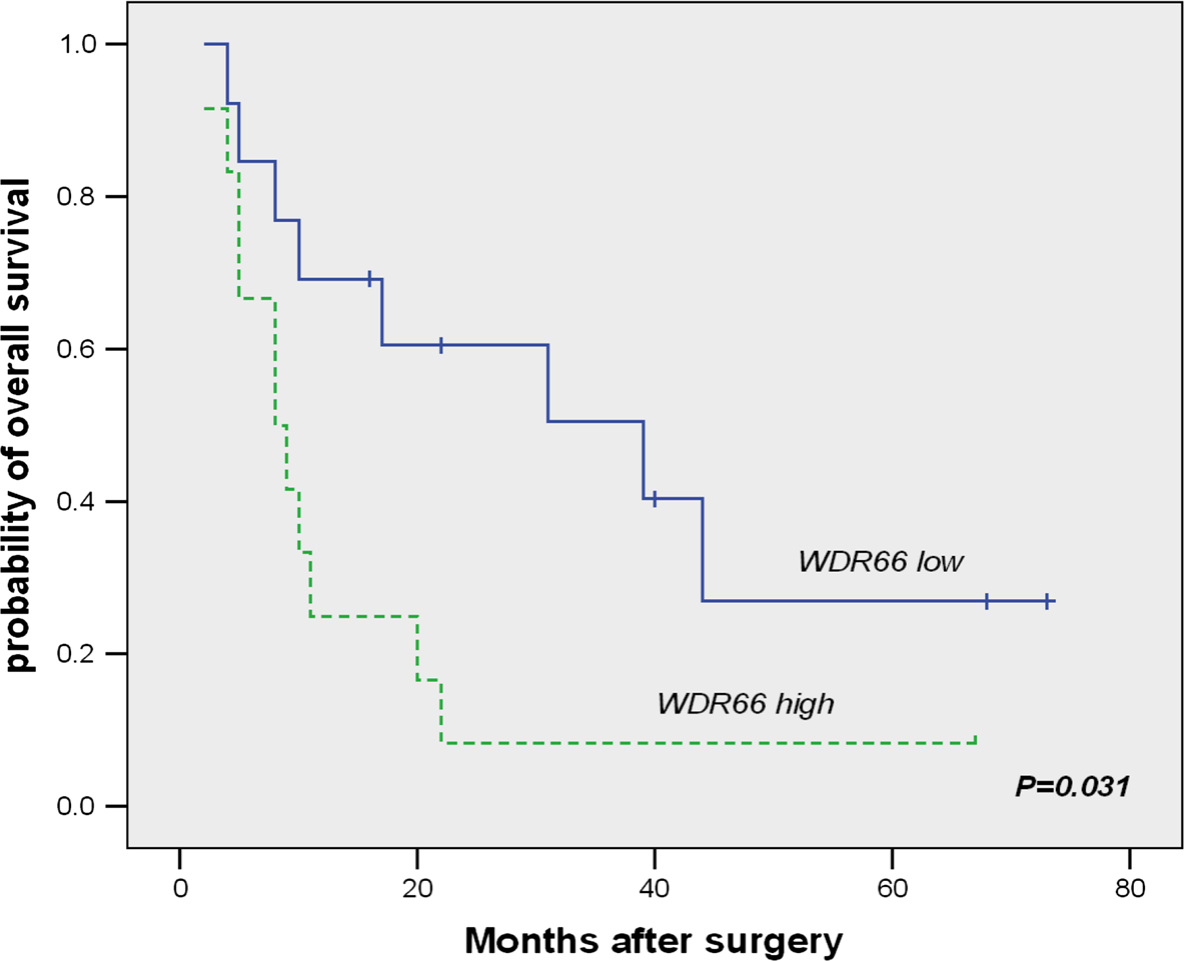
**High WDR66 mRNA expression is associated with poor survival in ESCC patients.** Kaplan-Meier analysis of survival of grouped according to WDR66 expression as measured by quantitative real-time RT-PCR. Grouping of patients according to median of qRT-PCR measurements was done as follows: WDR66 ≤ 125, WDR66 low (n = 12); WDR66 > 125, WDR66 high (n = 13). After choosing an optimal cut-point, analysis for WDR66 was done using maximally selected rank statistics. The group with patients expressing WDR66 at low levels showed a significantly better overall survival compared with the group with high levels of WDR66 expression (P = 0.031; log rank).

In order to test if WDR66 expression correlates with prognostic markers in a separate validation set of ESCC examples, we determined WDR66 expression in an independent set of n = 25 ESCC examples using qRT-PCR (Additional file 1: Table S1). High expression of WDR66 RNA was found to be a significant prognostic factor with regard to cancer-related survival (P = 0.031; Figure 2). When analyzed with regard to various clinicopathological parameters, such as gender (P = 0.804), age (P = 0.432), tumor differentiation (P =0.032), pT factor (P = 0.234), lymph node metastasis (P = 0.545), distant metastasis (P = 0.543) and TNM stage (P = 0.002; Table 1), multivariate Cox regression analysis revealed that WDR66 expression remained an independent prognostic factor (P = 0.042; Table 1).

**Table 1:** Cox regression analysis for factors possibly influencing disease-specific survival in patients with ESCC in our cohort

**Variables**	**Hazard ratio**	**95% CI**	***P***
**WDR66 (high vs low)**	**2.644**	**1.034-6.758**	**0.042**
**Gender (male vs female)**	**1.15**	**0.380-3.48**	**0.804**
**Age, y (<60 vs ≥60)**	**1.46**	**0.569-3.748**	**0.432**
**Tumor differentiation (high grade vs low or Intermediate grade)**	**2.763**	**1.093-6.983**	**0.032**
**pT factor ( pT3 or pT4 vs pT1 or pT2)**	**2.133**	**0.613-7.420**	**0.234**
**Lymph node metastasis (yes vs no)**	**1.575**	**0.362-6.852**	**0.545**
**Distant metastasis (yes vs no)**	**1.376**	**0.492-3.846**	**0.543**
**TNM stage (I or II vs III or IV)**	**7.711**	**2.181-27.259**	**0.002**

### Knockdown of WDR66 in KYSE520 effected VIM and OCLD expression in vitro

**Figure 3:**
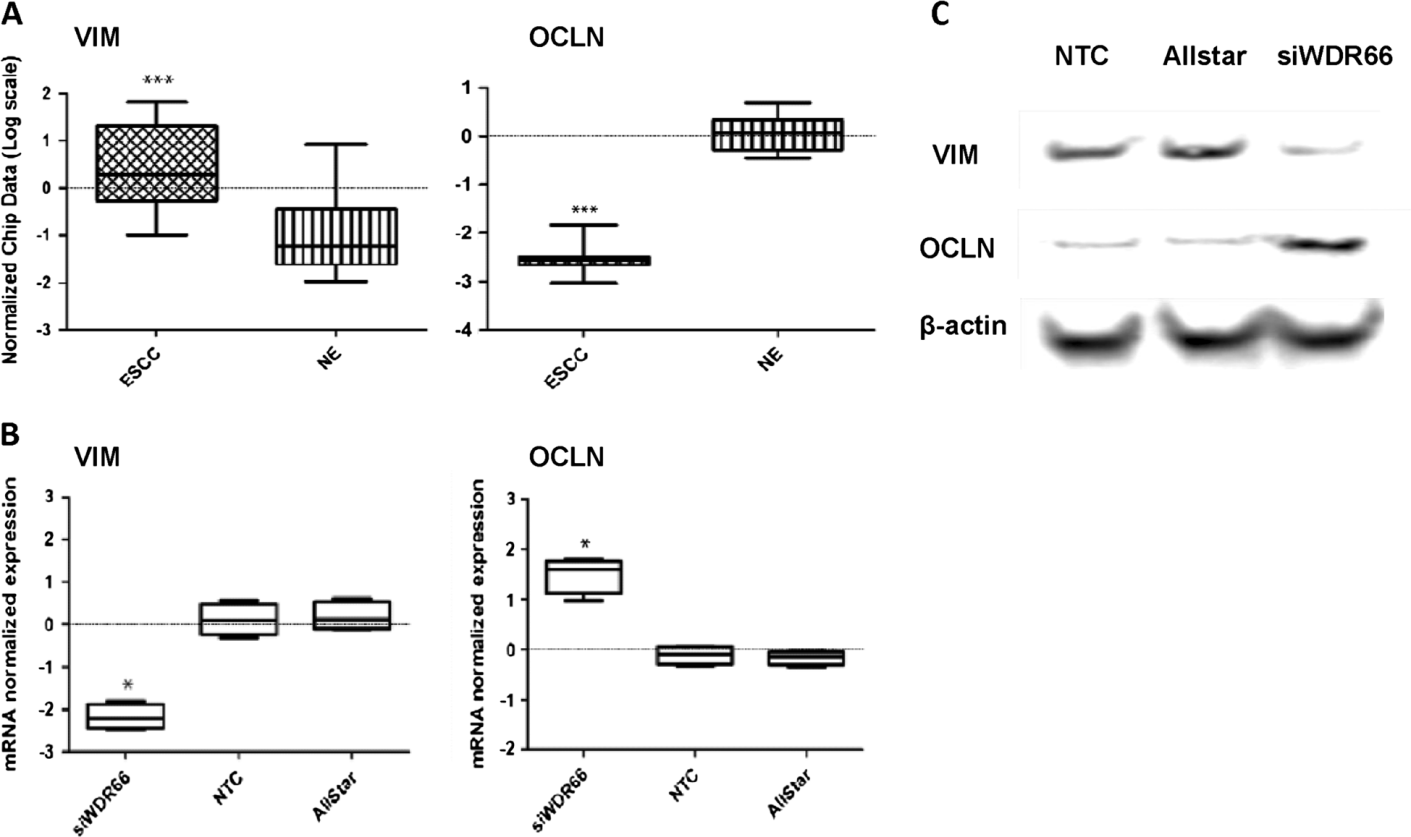
**Knockdown of WDR66 gene affects expression of vimentin and occludin. A**: mRNA expression of the VIM and OCLN gene in the original training cohort determined by microarray analysis. Array data analysis was performed on 18 healthy normal esophageal epithelium (NE) and 10 esophageal squamous cell carcinoma (ESCC) samples. Gene expression is presented as normalized (log2 scale) signal intensity of the genes of interest. VIM and OCLN are significantly differentially expressed in ESCC compared with NE (corrected p-value: VIM p = 0.0008; OCLD p < 0.0001). VIM expression level is low in NE but high in ESCC cases, whereas OCLN expression is high in NE but low in ESCC cases. The horizontal axis depicts the patient groups ESCC and NE. (∗∗∗ *P* < 0.001) **B**: Knockdown of WDR66 affects mRNA expression of VIM and OCLN. Gene expression is presented as normalized (log scale) signal intensity for mRNA expression levels of VIM and OCLN gene. Gene expression level of VIM was significantly down regulated whereas OCLN expression was significantly higher in cells treated with WDR66 siRNA in comparison to NTC (KYSE520) and Allstar (negative control siRNA) (corrected p-value: VIM p = 0.0286; OCLD p = 0.0186). Data are representatives of four independent experiments. (∗p < 0.05). **C**: Detection of vimentin and occludin protein by immunoblotting of KYSE520 cells treated with WDR66 siRNA in comparison to NTC (KYSE520) and Allstar (negative control siRNA). β-actin was used as loading control. Vimentin expression was significantly down regulated while occludin was significantly higher expressed in cells treated with WDR66 siRNA.

In order to learn more about the function of WDR66, RNA interference was used to silence its expression in KYSE520 cells, a human esophageal squamous cell carcinoma cell line which highly expressed WDR66. Subsequently, a microarray expression analysis was performed in order to identify the genes affected by WDR66 knockdown. A total of 699 genes was identified based on a two-fold change expression difference with p-value of p < 0.001. In an approach to link the observed gene expression profile to gene function, these 699 differentially expressed genes were subjected to gene ontology (GO) analysis. Functional enrichment analysis identified 10 GO terms to be significantly associated with the WDR66 knockdown (Additional file 2: Table S2). All these 10 GO terms are membrane related. We checked the expression of vimentin and occludin in ESCC patients of our cohort, and found that vimentin was highly expressed (p = 0.0008) while occludin was less expressed (p < 0.0001) in ESCC specimens in comparison to NE (Figure 3A). Microarray data were validated by qRT-PCR and Western blotting providing independent evidence of the changes of vimentin (VIM) and occludin (OCLD) expression associated with the WDR66 knockdown (Figure 3B, 3C).

### Knockdown of WDR66 in KYSE520 cells affects cell motility and results in growth suppression

**Figure 4:**
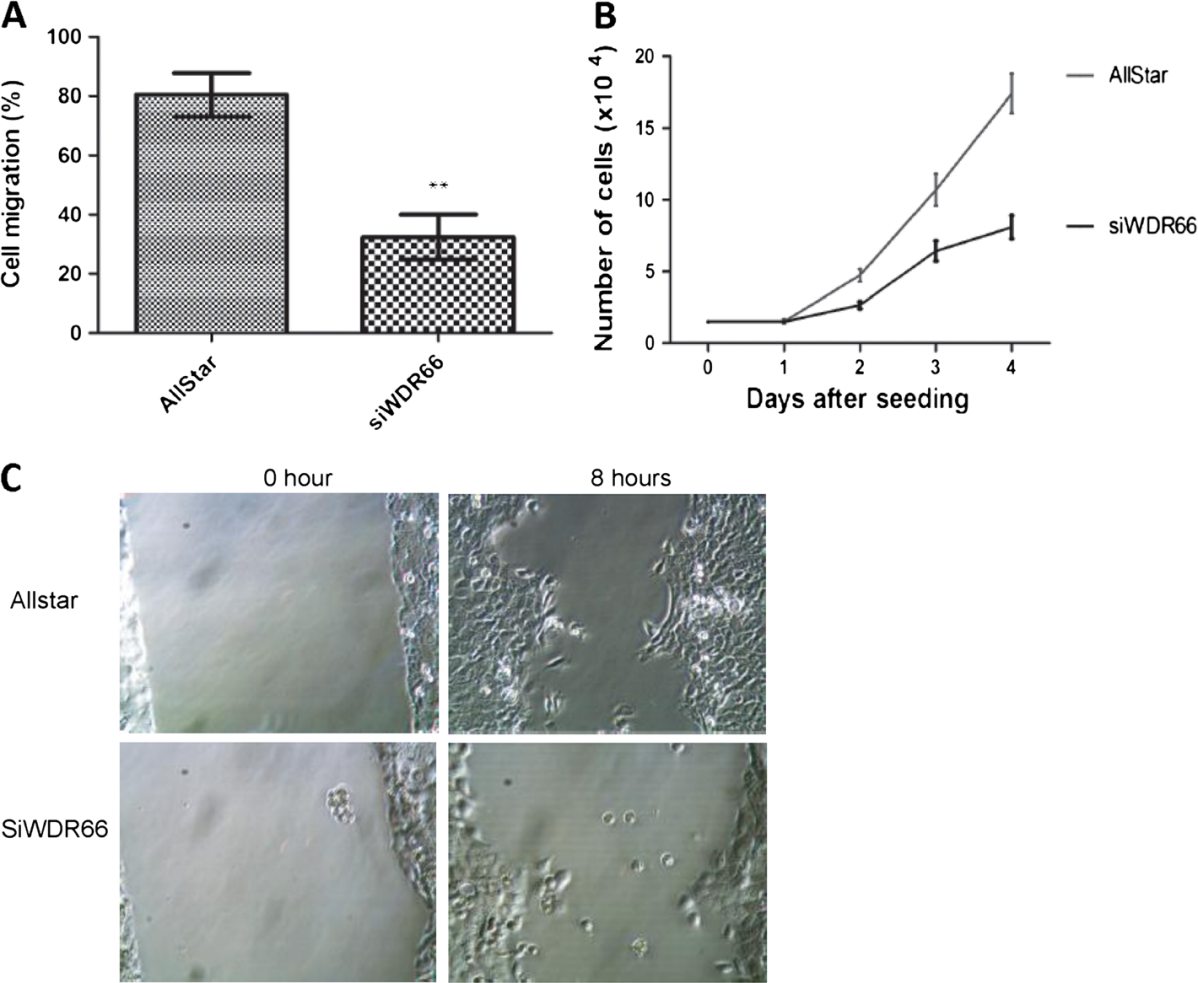
**Knockdown of WDR66 gene affects cell motility and cell growth. A**: Cell motility assays showed that knockdown of WDR66 reduced cell migration after 16 hours. About 35% of the siWDR66 cells were migrated in comparison to that of the mock control cells. The differences between siWDR66 and Allstar (negative control siRNA) cells were significant (p = 0.0032) using the paired t test. The data shown are representatives of three independent experiments and each done in quadruplicate. **B**: Knockdown of WDR66 leads to suppression of cell growth. A total of 1.5×10^4^ cells were seeded at day 0. WDR66 knockdown cells grew slower than cells treated with Allstar (negative control siRNA). The differences between siWDR66 and Allstar (negative control siRNA) treated cells were highly significant (p = 0. 0098). **C**: Wound-healing assays show that knockdown of WDR66 reduces cell motility. Representative images are shown. Images are taken immediately after insert was removed and 8 hours later. Original magnification is x400.

Due to the effect of WDR66 on the expression of vimentin, an important EMT marker which plays a central role in the reversible trans-differentiation and occludin, an adhesion molecules that is a constituent of tight junctions, we hypothesized that WDR66 may regulate cell motility of esophageal squamous cancer cells. WDR66 was silenced in the human squamous cell carcinoma cell line KYSE520 by RNA interference. Transfection efficiency was evaluated by qPCR. Cell migration assays showed that KYSE520 cells, which had been transfected with WDR66 siRNA, displayed a motility capacity of only 40% compared to the cells having been transfected with control siRNA (AllStar) after 16 hours (Figure 4A). Moreover, we found that introduction of siWDR66 remarkably suppressed growth of KYSE520 in comparison to control cells (Figure 4B). In order to visualize the involvement of WDR66 in cell migration and proliferation, a wound-healing assay was carried out. The insert was removed at defined time points of scratching and the results were recorded by taking pictures (Figure 4C). Thus, our data suggest that WDR66 promotes cell proliferation and affects cell motility.

## Discussion

By whole genome-wide expression profiling we found that WD repeat-containing protein 66 (WDR66) might be a useful biomarker for risk stratification and a modulator for epithelial-mesenchymal transition of ESCC. Among 20 normal human tissues examined by qRT-PCR, WDR66 was most abundantly expressed in the testis. Thus, our data suggest that WDR66 might be a cancer / testis antigen. Cancer-testis (CT) genes, normally expressed in germ line cells but also activated in a wide range of cancer types, often encode antigens in cancer patients [17]. Testis is an immune-privileged site as a result of a blood barrier and lack of HLA class I expression on the surface of germ cells [18]. Hence, if testis-specific genes are expressed in other tissues, they can be immunogenic. Expression of some cancer-testis genes in a high percentage of esophageal tumors makes them potential targets for immunotherapy, like LAGE1, MAGE-A4 and NY-ESO-1 [19]. NY-ESO-1 is a highly immunogenic, prototypical protein marker limited in expression to a wide variety of cancer types but not in normal tissue, with the exception of the immune-privileged testes, and has been heavily investigated as target for immunotherapy in cancer patients. Recently, immunotherapy of various cancers again NY-ESO-1 showed promising clinical results [20-22]. Unfortunately, most cancer / testis antigens are expressed only in a small group of tumor patients, among 10-40%. However, we found that the expression of WDR66 was specifically enhanced in 96% of ESCC patients and also very low or absent in normal tissues. WDR66 may be a novel Immunotherapy target for ESCC.

Our experiments revealed a strong association of WDR66 expression with vimentin and occludin. Vimentin is an important mesenchymal marker and plays a central role for reversible trans-differentiation [23]. Tumor cells undergoing EMT have an increased the ability for detachment from the main tumor bulk, which is a crucial step for tumor dissemination and metastasis [24]. EMT is accompanied by a switch from keratin to vimentin expression [25]. Earlier studies also demonstrated a correlation between the reduction of tight junctions and tumor metastasis. Here, we found a decreased expression of occludin, an important tight junction protein. According to a recent study comparing the risk of metastasis in ESCC and EAC, ESCC is characterized by a more aggressive behavior and tendency for early metastasis [26]. Thus, we hypothesize that the elevated expression of WDR66 in ESCC may promote EMT through an up-regulation of vimentin expression and a down regulation of occludin and cohesion of the tumor tissue. At this point, we checked the expression of vimentin and occludin in ESCC patients of our cohort, and found that vimentin was highly expressed while occludin was less expressed in ESCC specimens in comparison to NE which supports our hypothesis. A recent study showed that low expression of the tight junction protein claudin-4 is associated with poor prognosis in ESCC [27]. This is also in agreement with the results of our microarray study, which showed that claudin-4 was significantly less expressed in ESCC and also remarkably over expressed after WDR66 knockdown (data not shown).

## Conclusions

In summary, we have identified WDR66 as a potential novel prognostic marker and promising target for ESCC. This result is based on our observations that (1) WDR66 is specifically highly expressed in esophageal squamous cell carcinoma and high WDR66 expression correlates with poor overall survival, (2) WDR66 regulates vimentin and occludin expression and plays a crucial role for EMT, and (3) knockdown of WDR66 suppresses cell growth and motility and decreases cell viability of ESCC cells. Therefore, we propose that WDR66 plays a major role in ESCC biology. Our functional data furthermore warrants further investigation of selective targeting of WDR66 as a novel drug target for ESCC treatment.

## Abbreviations

CRC: Colorectal cancers; EAC: Esophageal adenocarcinoma; ESCC: Esophageal squamous cell carcinoma; EMT: Epithelial to mesenchymal transition; GAC: Gastric adenocarcinoma; NE: Normal esophageal; OCLD: Occludin; qRT-PCR: Quantitative reverse transcription- polymerase chain reaction; siRNA: Small interfering RNA; VIM: Vimentin; WDR66: WD repeat-containing protein 66.

## Competing interests

The authors declare that they have no competing interests.

## Authors’ contributions

QW have made substantial contributions to conception and design, acquisition of data, analysis and interpretation of data; CM has been involved in data acquisition, data analysis and interpretation of data, drafting the manuscript and revising it critically for important intellectual content; and WK contributed in writing and revising the paper and has given final approval of the version to be published. All authors read and approved the final manuscript.

## Pre-publication history

The pre-publication history for this paper can be accessed here:

http://www.biomedcentral.com/1471-2407/13/137/prepub

## Supplementary Material

Additional file 1: Table S1 The clinicopathologic characterization of the 25 ESCC Patients for survival analysis. Click here for file

Additional file 2: Table S2 Significantly enriched Gene Ontology (GO) terms identified for genes differentially expressed in siWDR66 Kyse520 cells. GO analysis was performed using GeneSpring. The 10 GO terms with the significant corrected P-value (FDR false discovery rate corrected for multiple testing) are depicted sorted by p-Value (noncorrected). Click here for file

## References

[B1] Siegel R Naishadham D Jemal A Cancer statistics, 2012 CA Cancer J Clin 2012 62 10 29 10.3322/caac.20138 22237781

[B2] Chen Y Fu D Xi J Ji Z Liu T Ma Y Zhao Y Dong L Wang Q Shen X Expression and clinical significance of UCH37 in human esophageal squamous cell carcinoma Dig Dis Sci 2012 57 2310 2317 10.1007/s10620-012-2181-9 22615012

[B3] Kausar T Ahsan A Hasan MR Lin L Beer DG Ralhan R Sperm protein 17 is a novel marker for predicting cisplatin response in esophageal squamous cancer cell lines Int J Cancer 2010 126 1494 1503 19685492 10.1002/ijc.24828

[B4] Qin YR Tang H Xie F Liu H Zhu Y Ai J Chen L Li Y Kwong DL Fu L Guan XY Characterization of tumor-suppressive function of SOX6 in human esophageal squamous cell carcinoma Clin Cancer Res 2011 17 46 55 10.1158/1078-0432.CCR-10-1155 21084391

[B5] Yamasaki M Makino T Masuzawa T Kurokawa Y Miyata H Takiguchi S Nakajima K Fujiwara Y Matsuura N Mori M Doki Y Role of multidrug resistance protein 2 (MRP2) in chemoresistance and clinical outcome in oesophageal squamous cell carcinoma Br J Cancer 2011 104 707 713 10.1038/sj.bjc.6606071 21206495 PMC3049584

[B6] Fong HK Hurley JB Hopkins RS Miake-Lye R Johnson MS Doolittle RF Simon MI Repetitive segmental structure of the transducin beta subunit: homology with the CDC4 gene and identification of related mRNAs Proc Natl Acad Sci USA 1986 83 2162 2166 10.1073/pnas.83.7.2162 3083416 PMC323251

[B7] Li D Roberts R WD-repeat proteins: structure characteristics, biological function, and their involvement in human diseases Cell Mol Life Sci 2001 58 2085 2097 10.1007/PL00000838 11814058 PMC11337334

[B8] Venter JC Adams MD Myers EW Li PW Mural RJ Sutton GG Smith HO Yandell M Evans CA Holt RA The sequence of the human genome Science 2001 291 1304 1351 10.1126/science.1058040 11181995

[B9] Frydman J Nimmesgern E Ohtsuka K Hartl FU Folding of nascent polypeptide chains in a high molecular mass assembly with molecular chaperones Nature 1994 370 111 117 10.1038/370111a0 8022479

[B10] Kudo Y Guardavaccaro D Santamaria PG Koyama-Nasu R Latres E Bronson R Yamasaki L Pagano M Role of F-box protein betaTrcp1 in mammary gland development and tumorigenesis Mol Cell Biol 2004 24 8184 8194 10.1128/MCB.24.18.8184-8194.2004 15340078 PMC515055

[B11] Silva FP Hamamoto R Nakamura Y Furukawa Y WDRPUH, a novel WD-repeat-containing protein, is highly expressed in human hepatocellular carcinoma and involved in cell proliferation Neoplasia 2005 7 348 355 10.1593/neo.04544 15967112 PMC1501145

[B12] Meisinger C Prokisch H Gieger C Soranzo N Mehta D Rosskopf D Lichtner P Klopp N Stephens J Watkins NA A genome-wide association study identifies three loci associated with mean platelet volume Am J Hum Genet 2009 84 66 71 10.1016/j.ajhg.2008.11.015 19110211 PMC2668036

[B13] Akdi A Gimenez EM Garcia-Quispes W Pastor S Castell J Biarnes J Marcos R Velazquez A WDR3 gene haplotype is associated with thyroid cancer risk in a Spanish population Thyroid 2010 20 803 809 10.1089/thy.2010.0072 20578902

[B14] Sato N Koinuma J Fujita M Hosokawa M Ito T Tsuchiya E Kondo S Nakamura Y Daigo Y Activation of WD repeat and high-mobility group box DNA binding protein 1 in pulmonary and esophageal carcinogenesis Clin Cancer Res 2010 16 226 239 10.1158/1078-0432.CCR-09-1405 20028748

[B15] Kemmner W Kessel P Sanchez-Ruderisch H Moller H Hinderlich S Schlag PM Detjen K Loss of UDP-N-acetylglucosamine 2-epimerase/N-acetylmannosamine kinase (GNE) induces apoptotic processes in pancreatic carcinoma cells FASEB J 2012 26 938 946 10.1096/fj.11-186700 22049060

[B16] Kretschmer C Sterner-Kock A Siedentopf F Schoenegg W Schlag PM Kemmner W Identification of early molecular markers for breast cancer Mol Cancer 2011 10 15 10.1186/1476-4598-10-15 21314937 PMC3045364

[B17] Hofmann O Caballero OL Stevenson BJ Chen YT Cohen T Chua R Maher CA Panji S Schaefer U Kruger A Genome-wide analysis of cancer/testis gene expression Proc Natl Acad Sci USA 2008 105 20422 20427 10.1073/pnas.0810777105 19088187 PMC2603434

[B18] Ghafouri-Fard S Modarressi MH Expression of cancer-testis genes in brain tumors: implications for cancer immunotherapy Immunotherapy 2012 4 59 75 10.2217/imt.11.145 22150001

[B19] Forghanifard MM Gholamin M Farshchian M Moaven O Memar B Forghani MN Dadkhah E Naseh H Moghbeli M Raeisossadati R Abbaszadegan MR Cancer-testis gene expression profiling in esophageal squamous cell carcinoma: identification of specific tumor marker and potential targets for immunotherapy Cancer Biol Ther 2011 12 191 197 10.4161/cbt.12.3.15949 21613820

[B20] Hunder NN Wallen H Cao J Hendricks DW Reilly JZ Rodmyre R Jungbluth A Gnjatic S Thompson JA Yee C Treatment of metastatic melanoma with autologous CD4+ T cells against NY-ESO-1 N Engl J Med 2008 358 2698 2703 10.1056/NEJMoa0800251 18565862 PMC3277288

[B21] Junqueira C Guerrero AT Galvao-Filho B Andrade WA Salgado AP Cunha TM Ropert C Campos MA Penido ML Mendonca-Previato L Trypanosoma cruzi adjuvants potentiate T cell-mediated immunity induced by a NY-ESO-1 based antitumor vaccine PLoS One 2012 7 e36245 10.1371/journal.pone.0036245 22567144 PMC3342165

[B22] Weide B Zelba H Derhovanessian E Pflugfelder A Eigentler TK Di Giacomo AM Maio M Aarntzen EH de Vries IJ Sucker A Functional T cells targeting NY-ESO-1 or Melan-A are predictive for survival of patients with distant melanoma metastasis J Clin Oncol 2012 30 1835 1841 10.1200/JCO.2011.40.2271 22529253

[B23] Vuoriluoto K Haugen H Kiviluoto S Mpindi JP Nevo J Gjerdrum C Tiron C Lorens JB Ivaska J Vimentin regulates EMT induction by Slug and oncogenic H-Ras and migration by governing Axl expression in breast cancer Oncogene 2011 30 1436 1448 10.1038/onc.2010.509 21057535

[B24] Kahlert C Lahes S Radhakrishnan P Dutta S Mogler C Herpel E Brand K Steinert G Schneider M Mollenhauer M Overexpression of ZEB2 at the invasion front of colorectal cancer is an independent prognostic marker and regulates tumor invasion in vitro Clin Cancer Res 2011 17 7654 7663 10.1158/1078-0432.CCR-10-2816 22042972

[B25] Mendez MG Kojima S Goldman RD Vimentin induces changes in cell shape, motility, and adhesion during the epithelial to mesenchymal transition FASEB J 2010 24 1838 1851 10.1096/fj.09-151639 20097873 PMC2874471

[B26] Gockel I Sgourakis G Lyros O Polotzek U Schimanski CC Lang H Hoppo T Jobe BA Risk of lymph node metastasis in submucosal esophageal cancer: a review of surgically resected patients Expert Rev Gastroenterol Hepatol 2011 5 371 384 10.1586/egh.11.33 21651355

[B27] Sung CO Han SY Kim SH Low expression of claudin-4 is associated with poor prognosis in esophageal squamous cell carcinoma Ann Surg Oncol 2011 18 273 281 10.1245/s10434-010-1289-4 20839069

