# Supplementary material for: Wdr66 is a novel marker for risk stratification and involved in epithelial-mesenchymal transition of esophageal squamous cell carcinoma
Source: BMC Cancer. 2013 Mar 21;13:137. doi: 10.1186/1471-2407-13-137 (PMC3610187; doi:10.1186/1471-2407-13-137)
Supplement: Additional file 1: Table S1 — The clinicopathologic characterization of the 25 ESCC Patients for survival analysis. [file 1471-2407-13-137-S1.docx]

| Number | | Sex | Age | Tumor Grade | pT | pN | M |
| --- | --- | --- | --- | --- | --- | --- | --- |
| 1 | | male | 58 | 2 | 3 | yes | no |
| 2 | | male | 49 | 2 | 3 | yes | no |
| 3 | | male | 58 | 3 | 3 | no | no |
| 4 | | male | 50 | 3 | 3 | no | no |
| 5 | | male | 67 | 3 | 3 | yes | no |
| 6 | | female | 58 | 2 | 1 | no | no |
| 7 | | female | 71 | 2 | 3 | yes | no |
| 8 | | male | 43 | 2 | 3 | yes | no |
| 9 | | male | 61 | 3 | 3 | yes | no |
| 10 | | male | 67 | 3 | 3 | yes | no |
| 11 | | male | 56 | 2 | 1 | yes | no |
| 12 | | female | 66 | 3 | 3 | yes | no |
| 13 | | male | 62 | 3 | 3 | yes | no |
| 14 | | male | 74 | 3 | 1 | yes | no |
| 15 | | female | 58 | 2 | 3 | yes | yes |
| 16 | | male | 54 | 2 | 4 | yes | yes |
| 17 | | male | 53 | 3 | 4 | yes | yes |
| 18 | | male | 60 | 2 | 3 | yes | no |
| 19 | | male | 58 | 3 | 3 | yes | yes |
| 20 | | male | 67 | 4 | 2 | yes | yes |
| 21 | | male | 53 | 2 | 3 | yes | no |
| 22 | | male | 64 | 3 | 3 | yes | no |
| 23 | | male | 64 | 3 | 1 | yes | no |
| 24 | | female | 50 | 3 | 3 | yes | no |
| 25 | | male | 43 | 3 | 3 | yes | no |
|  | | | | | | |  |

Supplementary table 1. The clinicopathologic characterization of the 25 ESCC Patients for survival analysis.
