# Supplementary material for: Wdr66 is a novel marker for risk stratification and involved in epithelial-mesenchymal transition of esophageal squamous cell carcinoma
Source: BMC Cancer. 2013 Mar 21;13:137. doi: 10.1186/1471-2407-13-137 (PMC3610187; doi:10.1186/1471-2407-13-137)
Supplement: Additional file 2: Table S2 — Significantly enriched Gene Ontology (GO) terms identified for genes differentially expressed in siWDR66 Kyse520 cells. GO analysis was performed using GeneSpring. The 10 GO terms with the significant corrected P-value (FDR false discovery rate corrected for multiple testing) are depicted sorted by p-Value (noncorrected). [file 1471-2407-13-137-S2.docx]

**Supplementary table 2. Significantly enriched Gene Ontology (GO) terms identified for genes differentially expressed in siWDR66 Kyse520 cells.** GO analysis was performed using GeneSpring. The 10 GO terms with the significant corrected P-value (FDR false discovery rate corrected for multiple testing) are depicted sorted by p-Value (noncorrected)
